# LncRNA UCA1, miR‐26a, and miR‐195 in coronary heart disease patients: Correlation with stenosis degree, cholesterol levels, inflammatory cytokines, and cell adhesion molecules

**DOI:** 10.1002/jcla.24070

**Published:** 2021-12-01

**Authors:** Jie Li, Zhisong Chen, Xiaoyan Wang, Haoming Song

**Affiliations:** ^1^ Department of Cardiology Tongji Hospital, School of Medicine, Tongji University Shanghai China; ^2^ Department of Cardiology Hospital Affiliated of Jiangnan University Wuxi China

**Keywords:** coronary heart disease, inflammatory cytokines and cell adhesion molecules, long noncoding RNA urothelial cancer‐associated 1, microRNA‐26a and microRNA‐195, stenosis degree

## Abstract

**Background:**

Long noncoding RNA urothelial cancer‐associated 1 (lnc‐UCA1) targets microRNA‐26a (miR‐26a) and microRNA‐195 (miR‐195) to participate in coronary heart disease (CHD) progression via regulation of vascular smooth muscle cell and microvascular endothelial cell viability and mobility. Therefore, this study set out to further explore the relationship between lnc‐UCA1 and miR‐26a and miR‐195, along with their roles in the management of patients with CHD.

**Methods:**

One hundred and thirty‐six CHD patients and 70 age‐/gender‐matched controls were recruited in this case‐control study. Their peripheral blood mononuclear cell samples were collected for lnc‐UCA1, miR‐26a, and miR‐195 measurement. Furthermore, serum samples from CHD patients were obtained for inflammatory cytokines and cell adhesion molecules measurement. The Gensini score was used to evaluate the stenosis severity in CHD patients.

**Results:**

Lnc‐UCA1 expression tend to be increased, while miR‐26a and miR‐195 expressions were reduced in patients with CHD compared to that of controls (all *p *< 0.001). In CHD patients, lnc‐UCA1 was negatively correlated with miR‐26a (*p *< 0.001) and miR‐195 (*p *= 0.014). Besides, lnc‐UCA1 was positively correlated with Gensini score (*p *< 0.001), total cholesterol (*p *= 0.019), low‐density lipoprotein cholesterol (*p *= 0.002), and C‐reactive protein (*p *< 0.001), while miR‐26a (*p *< 0.001) and miR‐195 (*p *= 0.002) were negatively correlated with Gensini score. What's more, lnc‐UCA1 was positively correlated with tumor necrosis factor (TNF)‐α (*p *= 0.004), interleukin (IL)‐1β (*p *= 0.041), vascular cell adhesion molecule‐1 (VCAM‐1) (*p *= 0.010), and intercellular adhesion molecule‐1 (ICAM‐1) (*p *< 0.001). While miR‐26a was negatively correlated with some of the individual inflammatory cytokines and cell adhesion molecules.

**Conclusion:**

Lnc‐UCA1, miR‐26a, and miR‐195 may serve as potential biomarkers for CHD management.

## Graphical abstract

There were 136 coronary heart disease (CHD) patients and 70 age‐/gender‐matched controls constitutively recruited. Their peripheral blood mononuclear cell samples were collected for long noncoding RNA urothelial cancer‐associated 1 (lnc‐UCA1), microRNA‐26a (miR‐26a), and microRNA‐195 (miR‐195) measurement. Besides, serum samples from CHD patients were obtained for inflammatory cytokines and cell adhesion molecules measurement. Lnc‐UCA1 expression was increased, while miR‐26a and miR‐195 expressions were reduced in CHD patients compared to controls. In CHD patients, lnc‐UCA1 negatively correlated with miR‐26a and miR‐195. Besides, lnc‐UCA1 positively correlated, while miR‐26a and miR‐195 negatively correlated with Gensini score. Moreover, lnc‐UCA1 positively correlated with total cholesterol (TC), low‐density lipoprotein cholesterol (LDL‐C), C‐reactive protein (CRP), tumor necrosis factor (TNF)‐α, interleukin (IL)‐1β, vascular cell adhesion molecule‐1 (VCAM‐1), and intercellular adhesion molecule‐1 (ICAM‐1), while miR‐26a negatively correlated with TNF‐α, IL‐6, VCAM‐1, and ICAM‐1. Also, miR‐195 negatively correlated with IL‐1β and ICAM‐1. These findings suggest that lnc‐UCA1, miR‐26a, and miR‐195 may serve as potential biomarkers for CHD patients’ management.

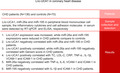

## 1 INTRODUCTION

Coronary heart disease (CHD) is the leading cause of death globally characterized by the presence of atherosclerotic plaque resulting in flow‐limiting obstruction in coronary arteries.^1^, ^2^ Of note, CHD is responsible for 9.1 million deaths in 2019 worldwide with a higher proportion of males than females.^3^ To the best of our knowledge, although effective treatment has been applied in CHD patients (such as thrombolytic therapy, percutaneous coronary intervention (PCI) therapy, and coronary artery bypass surgery etc.), recurrence and disease progression are prevalent among CHD patients who require readmission and intensive care leading to unfavorable prognosis.^4^, ^5^, ^6^, ^7^, ^8^ Hence, it is necessary to identify the potential biomarkers to monitor disease progression and further to individualize CHD management.

Long noncoding RNAs (lncRNAs) have been reported to be highly involved in cardiovascular biology and diseases.^9^, ^10^, ^11^ Originally identified as an oncogene in urothelial carcinoma, lncRNA urothelial cancer‐associated 1 (lnc‐UCA1) also participates in cardiovascular disease pathogenesis by regulating proliferation and migration of microvascular environment cells as well as mediating oxidative stress and mitochondrial function of macrophage from the recent researches.^12^, ^13^, ^14^, ^15^, ^16^, ^17^ Specifically, lnc‐UCA1 targets microRNA‐26a (miR‐26a) and microRNA‐195 (miR‐195) to promote proliferation and migration in vascular smooth muscle cells (VSMCs) and microvascular endothelial cells, respectively.^15^, ^16^ Also, miR‐26a and miR‐195 are two well‐established microRNAs with protective roles in cardiovascular diseases by regulating proliferation, migration, and invasion of vasculature‐related cell (such as endothelial cell and VSMCs).^18^, ^19^, ^20^, ^21^ While few studies report the roles of lnc‐UCA1, miR‐26a, and miR‐195 in cardiovascular disease patients especially in CHD patients, not to mention the intercorrelation between lnc‐UCA1 and miR‐26a and miR‐195 in these patients.^22^, ^23^ In our preliminary study with a relatively small sample size, we observed an elevation of lnc‐UCA1 expression in CHD patients compared to controls.

Therefore, this study aimed to explore the relationship between lnc‐UCA1 and miR‐26a and miR‐195, as well as their clinical value in CHD patients’ management.

## 2 METHODS

### 2.1 Subjects

This was a case‐control study. Between January 2018 and July 2020, this study consecutively enrolled 136 patients who were confirmed as CHD by coronary angiography (CAG) due to unexplained chest pain or suspected CHD symptoms in our hospital. The enrollment criteria were as follows: (i) diagnosed as CHD which was based on typical angina symptom and confirmed by CAG (at least one major epicardial vessel with >50% stenosis); (ii) ages older than 18 years; (iii) willing to provide peripheral blood (PB) samples for study analysis. The patients were excluded from the study if they had the conditions as follows: (i) complicated with inflammatory diseases, autoimmune disease or severe infections; (ii) received cardiac surgery, including open‐heart surgery and minimally invasive surgery before recruitment; (iii) had history of cancers or malignancies; (iv) during pregnancy or lactation. At the same time, another 70 subjects with matched age and gender to CHD patients were also enrolled in the study as controls. During the enrollment, the controls were restricted in 40–80 years old, and the sex ratio of controls was limited as 4:1 (male:female). All controls presented with symptoms of unexplained chest pain or suspected CHD symptoms at admission, then were excluded from CHD by CAG examination. Controls were ineligible for recruitment if they were pregnant and lactating women, concomitant with autoimmune disease, inflammatory diseases, severe infections, or had history of cancers or malignancies. This study was approved by the Institutional Review Board of Tongji Hospital Affiliated to Tongji University with approval number 2018‐LCYJ‐026. All subjects signed the informed consents.

### 2.2 Data recording and sample collection

After enrollment, clinical data of all subjects were recorded, including age, gender, body mass index (BMI), smoke, family history of CHD, comorbidities, and biochemical indexes. Besides, Gensini score was used to quantify the degree of coronary artery stenosis.^24^ Gensini score was the sum of lesion scores which were calculated by multiplying the stenosis degree score by the severity coefficient of lesion segment. A higher Gensini score indicated a more severe coronary artery stenosis. For sample collection, PB was sampled from all subjects before CAG. Sequentially, peripheral blood mononuclear cells (PBMCs) were separated from PB samples using Ficoll‐Hypaque density gradient centrifugation at 18℃, 1500 revolutions per minute for 30 min, and serum was isolated from PB samples using centrifuge.^25^ The PBMCs and serum were stored at −80 and 4℃ for necessary biochemical tests and study determination, respectively.

### 2.3 Reverse transcription‐quantitative polymerase chain reaction (RT‐qPCR) assay

PBMCs of all subjects were used to determine the expression of lnc‐UCA1, miR‐26a, and miR‐195 by RT‐qPCR. In brief, total RNA extraction was conducted using QIAamp RNA Blood Mini Kit (Qiagen). Then, reverse transcription was achieved using PrimeScript™ RT reagent Kit (Perfect Real Time) (Takara). Subsequently, the qPCR reaction was performed by QuantiNova SYBR Green PCR Kit (Qiagen). The relative expression of lnc‐UCA1, miR‐26a, and miR‐195 was calculated using 2^−ΔΔCt^method using GAPDH as the internal reference for lnc‐UCA1, and U6 as the internal reference for miR‐26a and miR‐195. The designed PCR primer sequence was referred to previous studies.^26^, ^27^, ^28^

### 2.4 Enzyme‐linked immunosorbent assay (ELISA)

Subsequently, the inflammatory cytokines, including tumor necrosis factor alpha (TNF‐α), interleukin‐1β (IL‐1β), and interleukin‐6 (IL‐6), and the cell adhesion molecules, including vascular cell adhesion molecule‐1 (VCAM‐1) and intercellular adhesion molecule 1 (ICAM‐1), in serum samples of CHD patients were determined by ELISA. All ELISA kits were purchased from Bio‐Techne China Co., Ltd. (R&D Systems). Assay was carried out referring to complete assay protocol recommended by the manufacturer. Briefly, 100 µl of assay diluent and 50 µl of standards, control, or sample were added to every well, which was incubated for 2 h. Then, every well was washed for four times, and 100 µl of conjugate was added to every well, followed by incubation for 1 h and washing for four times. Following that, 200 µl of substrate solution was added to every well, followed by incubation at room temperature for 30 min avoiding light. Afterwards, 50 µl of stop solution was added to every well, and absorbance at 450 nm was read immediately. Finally, standard curve was fitted, which was used for calculating the concentration of unknown samples.

### 2.5 Statistical analysis

Statistical analysis and graph plotting were severally completed using SPSS 24.0 (IBM Corp.) and GraphPad Prism 6.01 software (GraphPad Software Inc.). The mean and standard deviation (SD) were applied to describe the normally distributed variables, and the median with interquartile range (IQR) was used to expound the skewed‐distributed variables. Frequency was used for describing the categorized variables. Comparisons between CHD patients and controls were evaluated using Student's *t* test, Mann‐Whitney *U* test, and Chi‐square test. The performance of lnc‐UCA1/miR‐26a/miR‐195 expressions in distinguishing CHD patients from controls was estimated using receiver‐operating characteristic (ROC) curve analysis. Associations between two continuous variables were checked by Spearman rank correlation test. Forward multivariate logistic regression analysis was conducted to identify the potential factor relating to CHD risk. A *p* value less than 0.05 indicated statistical significance.

## 3 RESULTS

### 3.1 Patients’ clinical characteristics

The CHD patients presented with a mean age of 62.6 ± 9.5 years, which consisted of 25 (18.4%) females and 111 (81.6%) males (Table 1), while controls exhibited a mean age of 61.4 ± 6.8 years, consisting of 18 (25.7%) female and 52 (74.3%) male participants. No difference of clinical characteristics, including age (*p *= 0.305) or gender (*p *= 0.220) between CHD patients and controls. Although CHD patients displayed an increased proportion of diabetes mellitus (25.0% vs. 12.9%, *p *= 0.042), elevated C‐reactive protein (CRP) level (9.1 (6.7–11.8) mg/L vs. 5.5 (2.4–10.2) mg/L, *p *< 0.001), and higher Gensini score (39.3 ± 25.0 vs. 1.3 ± 2.0, *p *< 0.001) compared to controls, majority of the clinical features were similar between these two groups (all *p *> 0.05) (Table 1).

**TABLE 1:** Characteristics of CHD patients and controls

Items	Controls (*N* = 70)	CHD patients (*N* = 136)	*p* value
Age (years), mean ± SD	61.4 ± 6.8	62.6 ± 9.5	0.305
Gender, No. (%)			0.220
Female	18 (25.7)	25 (18.4)	‐
Male	52 (74.3)	111 (81.6)	‐
BMI (kg/m^2^), mean ± SD	23.5 ± 3.0	23.9 ± 2.9	0.360
Smoke, No. (%)	24 (34.3)	63 (46.3)	0.098
Family history of CHD, No. (%)	13 (18.6)	34 (25.0)	0.298
Hypertension, No. (%)	50 (71.4)	103 (75.7)	0.503
Hyperlipidemia, No. (%)	29 (41.4)	72 (52.9)	0.117
Hyperuricemia, No. (%)	21 (30.0)	50 (36.8)	0.333
DM, No. (%)	9 (12.9)	34 (25.0)	0.042
FBG (mmol/L), median (IQR)	5.4 (4.9–5.9)	5.7 (5.1–6.3)	0.105
Scr (μmol/L), mean ± SD	75.3 ± 13.0	77.8 ± 15.7	0.254
SUA (μmol/L), median (IQR)	363.6 (316.6–403.3)	345.0 (308.9–388.9)	0.142
TG (mmol/L), median (IQR)	1.4 (0.9–2.0)	1.6 (0.9–2.3)	0.325
TC (mmol/L), mean ± SD	4.5 ± 1.0	4.7 ± 1.1	0.079
LDL‐C (mmol/L), mean ± SD	2.8 ± 0.6	3.0 ± 0.7	0.108
HDL‐C (mmol/L), mean ± SD	1.0 ± 0.3	0.9 ± 0.2	0.262
CRP (mg/L), median (IQR)	5.5 (2.4–10.2)	9.1 (6.7–11.8)	<0.001
Gensini score, mean ± SD	1.3 ± 2.0	39.3 ± 25.0	<0.001

Abbreviations: BMI, body mass index; CHD, coronary heart disease; CRP, C‐reactive protein; DM, diabetes mellitus; FBG, fasting blood glucose; HDL‐C, high‐density lipoprotein cholesterol; IQR, interquartile range; LDL‐C, low‐density lipoprotein cholesterol; Scr, serum creatinine; SD, standard deviation; SUA, serum uric acid; TC, total cholesterol; TG, triglyceride.

### 3.2 Lnc‐UCA1, miR‐26a, and miR‐195 expressions

**FIGURE 1:**
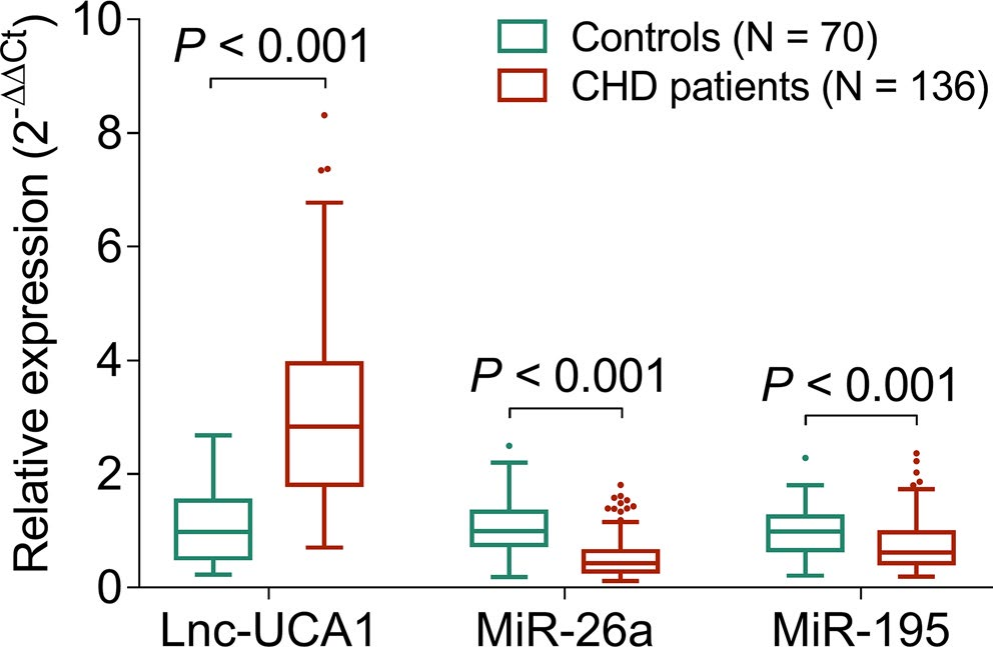
Comparison of lnc‐UCA1, miR‐26a, and miR‐195 expressions between CHD patients and controls. CHD, coronary heart disease; lnc‐UCA1, long noncoding RNA‐urothelial cancer‐associated 1; miR‐26a, microRNA‐26a; miR‐195, microRNA‐195

**FIGURE 2:**
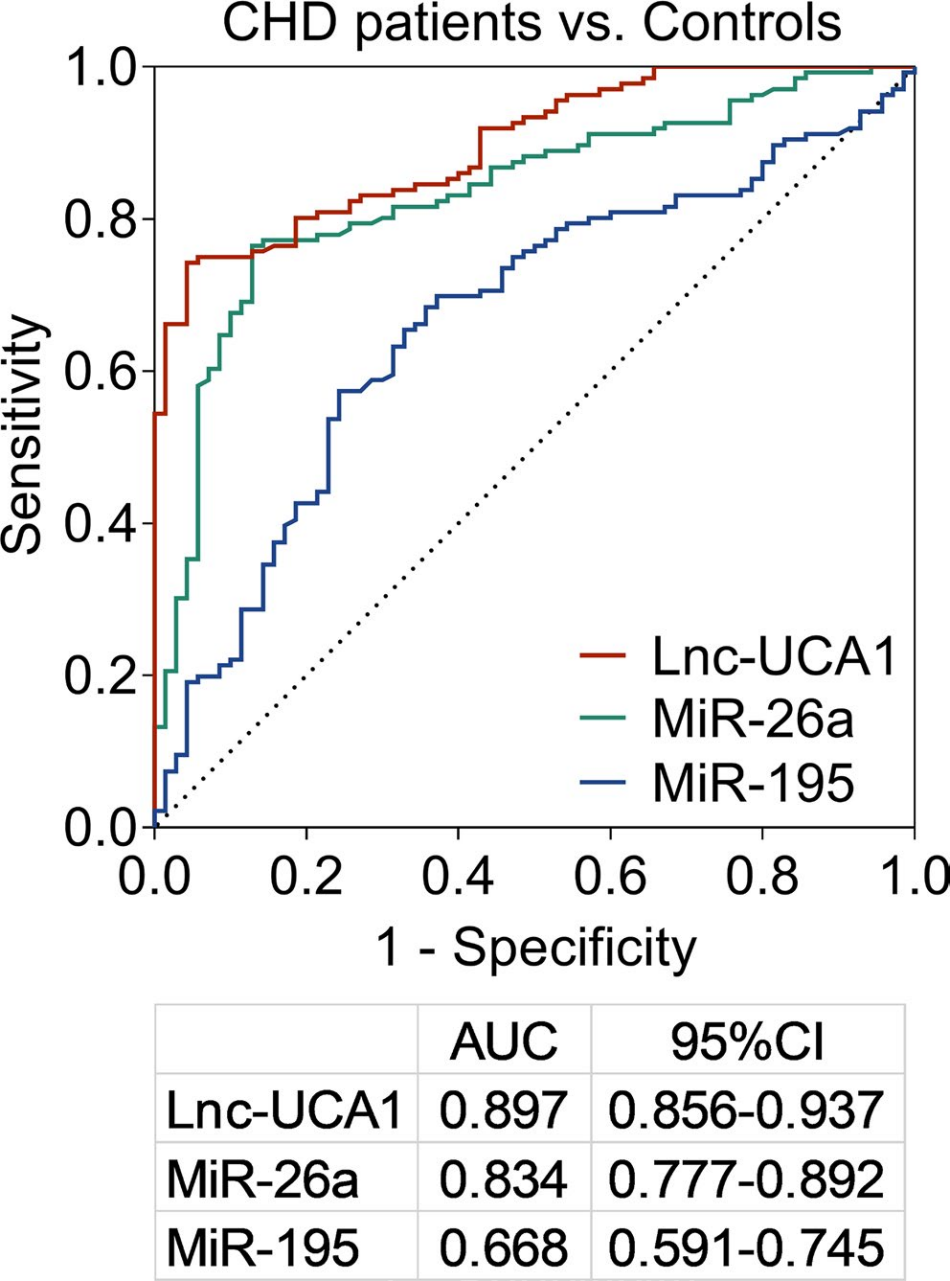
Lnc‐UCA1, miR‐26a, and miR‐195 correlated with CHD risk. AUC, area under curve; CI, confidence interval; Lnc‐UCA1, long noncoding RNA‐urothelial cancer‐associated 1; miR‐26a, microRNA‐26a; miR‐195, microRNA‐195

Lnc‐UCA1 expression tend to be increased, while miR‐26a and miR‐195 expressions were reduced in patients with CHD compared to that of controls (all *p *< 0.001, Figure 1). Further ROC curve analysis indicated that lnc‐UCA1 (area under curve (AUC): 0.897, 95% confidence interval (CI): 0.856–0.937, Figure 2), miR‐26a (AUC: 0.834, 95% CI: 0.777–0.892), and miR‐195 (AUC: 0.668, 95% CI: 0.591–0.745) all could distinguish the patients with CHD from controls, among which lnc‐UCA1 exhibited the highest AUC.

### 3.3 Correlation of lnc‐UCA1 with miR‐26a and miR‐195

**FIGURE 3:**
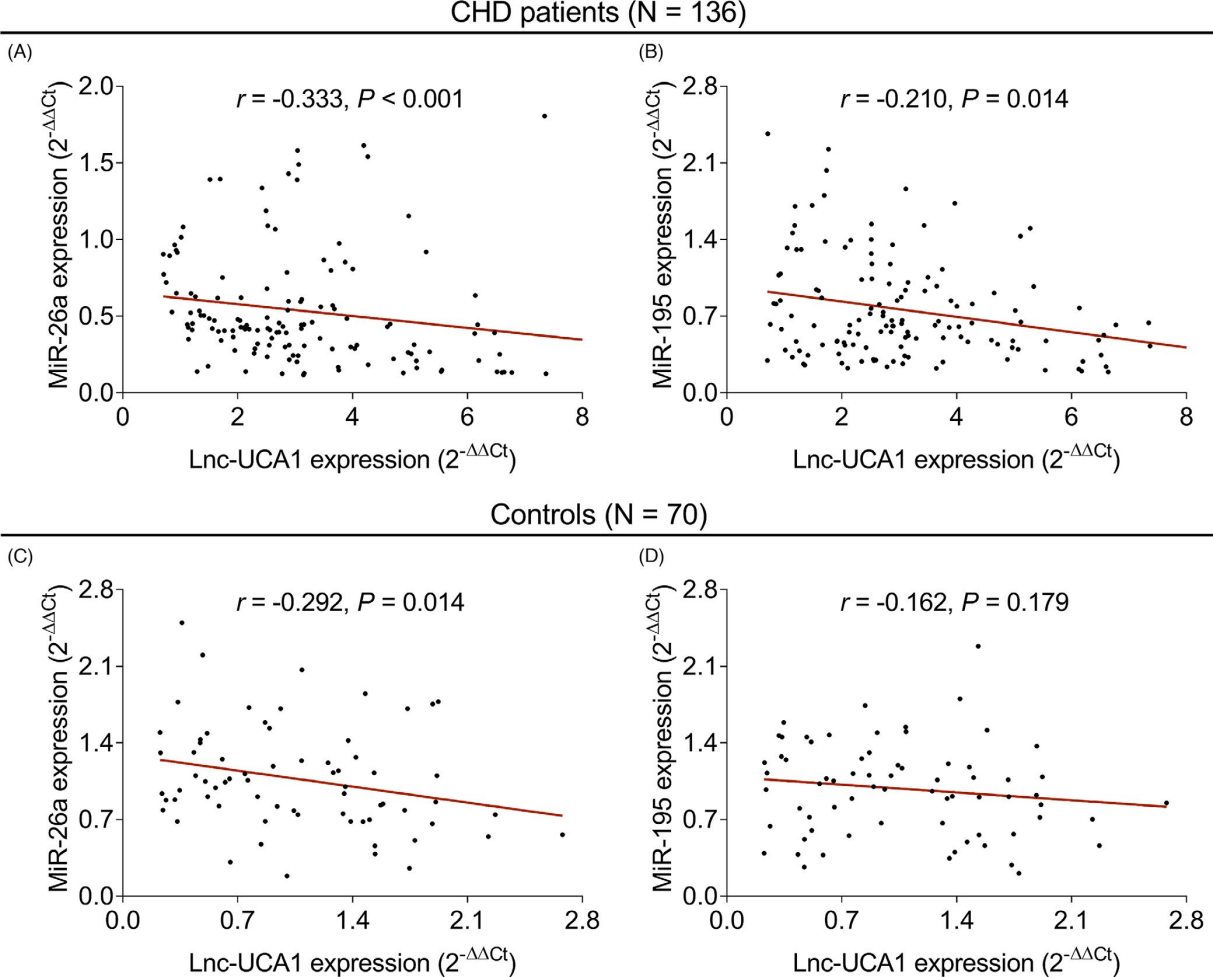
Lnc‐UCA1 was negatively correlated with miR‐26a and miR‐195 in CHD patients. Correlation of lnc‐UCA1 with miR‐26a (A) and miR‐195 (B) in CHD patients. Correlation of lnc‐UCA1 with miR‐26a (C) and miR‐195 (D) in controls. CHD, coronary heart disease; Lnc‐UCA1, long noncoding RNA‐urothelial cancer‐associated 1; miR‐26a, microRNA‐26a; miR‐195, microRNA‐195

In CHD patients, lnc‐UCA1 was negatively correlated with miR‐26a (*r *= −0.333, *p *< 0.001, Figure 3A) and miR‐195 (*r *= −0.210, *p *= 0.014, Figure 3B). However, in controls, lnc‐UCA1 was negatively associated with miR‐26a (*r *= −0.292, *p *< 0.001, Figure 3C), although it did not correlate with miR‐195 (*r *= −0.162, *p *= 0.179, Figure 3D).

### 3.4 Correlation of lnc‐UCA1, miR‐26a, and miR‐195 with stenosis degree of CHD patients

**FIGURE 4:**
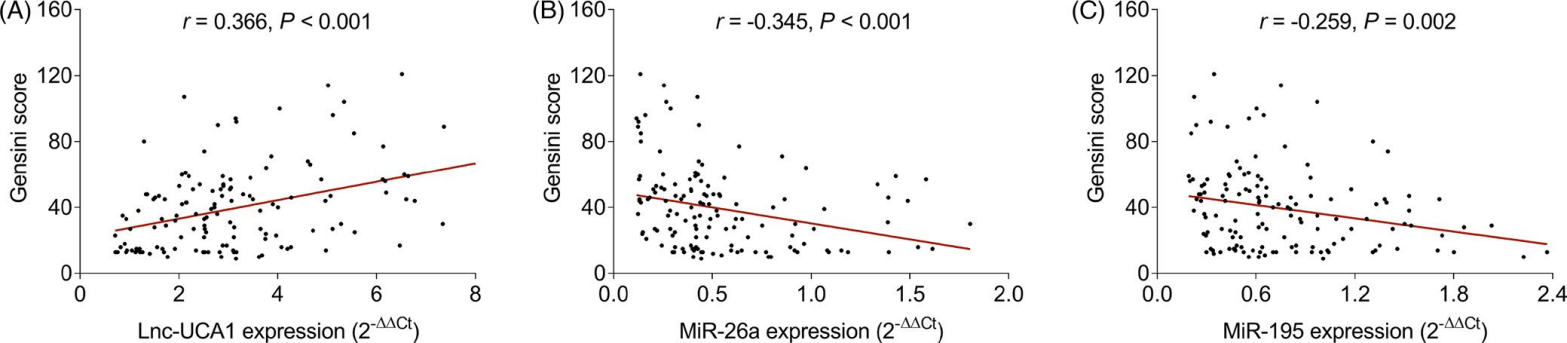
Lnc‐UCA1 was positively correlated, while miR‐26a and miR‐195 were negatively correlated with Gensini score. Correlation of lnc‐UCA1 (A), miR‐26a (B), and miR‐195 (C) with Gensini score in CHD patients. Lnc‐UCA1, long noncoding RNA‐urothelial cancer‐associated 1; miR‐26a, microRNA‐26a; miR‐195, microRNA‐195

Gensini score was used to assess the stenosis degree, then we discovered that lnc‐UCA1 was positively correlated (*r *= 0.366, *p *< 0.001, Figure 4A), while miR‐26a (*r *= −0.345, *p *< 0.001, Figure 4B) and miR‐195 (*r *= −0.259, *p *= 0.002, Figure 4C) were negatively correlated with Gensini score in the patients with CHD.

### 3.5 Correlation of lnc‐UCA1, miR‐26a, and miR‐195 with biochemical indexes in CHD patients

Lnc‐UCA1 was positively correlated with total cholesterol (TC) (*r *= 0.200, *p* = 0.019, Table 2), low‐density lipoprotein cholesterol (LDL‐C) (*r *= 0.266, *p* = 0.002), and CRP (*r *= 0.344, *p *< 0.001) in the patients with CHD. Moreover, miR‐26a was negatively correlated with triglyceride (TG) (*r *= −0.179, *p* = 0.037), TC (*r *= −0.177, *p* = 0.039), and CRP (*r *= −0.397, *p*<0.001); miR‐195 was negatively associated with TC (*r *= −0.196, *p* = 0.022) and LDL‐C (*r *= −0.186, *p* = 0.030) in the patients with CHD. Furthermore, there was no correlation of lnc‐UAC1, miR‐26a, or miR‐195 with other biochemical indexes in CHD patients as displayed in Table 2.

**TABLE 2:** Correlation of lnc‐UCA1, miR‐26a, and miR‐195 with routine blood biochemical indexes in CHD patients

Items	Lnc‐UCA1		MiR−26a		MiR−195	
	*r*	*p* value	*r*	*p* value	*r*	*p* value
FBG	−0.061	0.484	0.093	0.282	0.097	0.259
Scr	0.056	0.520	−0.036	0.681	−0.087	0.311
SUA	0.107	0.214	−0.162	0.060	−0.045	0.603
TG	0.095	0.273	−0.179	0.037	−0.086	0.321
TC	0.200	0.019	−0.177	0.039	−0.196	0.022
LDL‐C	0.266	0.002	−0.156	0.070	−0.186	0.030
HDL‐C	−0.136	0.115	0.038	0.659	−0.100	0.245
CRP	0.344	<0.001	−0.397	<0.001	−0.140	0.105

Abbreviations: CHD, coronary heart disease; CRP, C‐reactive protein; FBG, fasting blood glucose; HDL‐C, high‐density lipoprotein cholesterol; LDL‐C, low‐density lipoprotein cholesterol; Lnc‐UCA1, long noncoding RNA urothelial carcinoma‐associated 1; miR, microRNA; Scr, serum creatinine; SUA, serum uric acid; TC, total cholesterol; TG, triglyceride.

### 3.6 Correlation of lnc‐UCA1, miR‐26a, and miR‐195 with inflammatory cytokines in CHD patients

**FIGURE 5:**
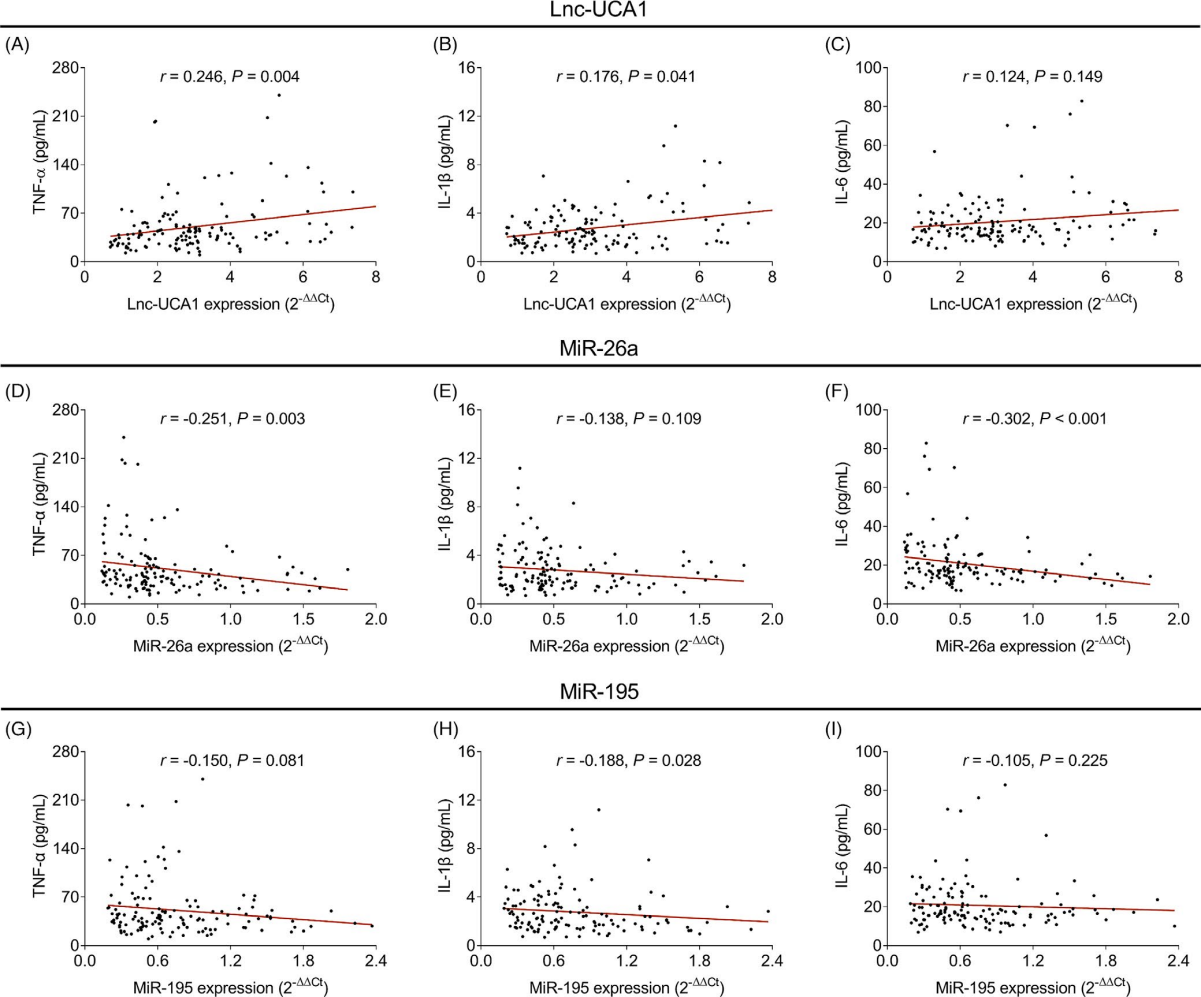
Lnc‐UCA1 was positively correlated, while miR‐26a and miR‐195 were negatively correlated with inflammatory cytokines. Correlation of lnc‐UCA1 with TNF‐α (A), IL‐1β (B), and IL‐6 (C) in CHD patients. Correlation of miR‐26a with TNF‐α (D), IL‐1β (E), and IL‐6 (F) in CHD patients. Correlation of miR‐195 with TNF‐α (G), IL‐1β (H), and IL‐6 (I) in CHD patients. CHD, coronary heart disease; IL‐1β, interleukin‐1β; IL‐6, interleukin‐6; Lnc‐UCA1, long noncoding RNA‐urothelial cancer‐associated 1; miR‐195, microRNA‐195; miR‐26a, microRNA‐26a; TNF‐α, tumor necrosis factor alpha

Lnc‐UCA1 was positively correlated with TNF‐α (*r *= 0.246, *p* = 0.004, Figure 5A) and IL‐1β (*r *= 0.176, *p* = 0.041, Figure 5B), while it did not correlate with IL‐6 (*r *= 0.124, *p* = 0.149, Figure 5C) in the patients with CHD. Besides, miR‐26a was negatively correlated with TNF‐α (*r *= −0.251, *p* = 0.003, Figure 5D) and IL‐6 (*r *= −0.302, *p *< 0.001, Figure 5F), but not correlated with IL‐1β (*r *= −0.138, *p* = 0.109, Figure 5E). Although no correlation of miR‐195 with TNF‐α (*r *= −0.150, *p* = 0.081, Figure 5G) or IL‐6 (*r *= −0.105, *p* = 0.225, Figure 5I) was observed, miR‐195 was negatively associated with IL‐1β (*r *= −0.188, *p* = 0.028, Figure 5H).

### 3.7 Correlation of lnc‐UCA1, miR‐26a, and miR‐195 with cell adhesion molecules in CHD patients

Lnc‐UCA1 was positively correlated with VCAM‐1 (*r *= 0.219, *p* = 0.010, Supplementary Figure S1A) and ICAM‐1 (*r *= 0.314, *p *< 0.001, Supplementary Figure S1B) in the patients with CHD. Moreover, miR‐26a was negatively correlated with VCAM‐1 (*r *= −0.263, *p* = 0.002, Supplementary Figure S1C) and ICAM‐1 (*r *= −0.304, *p *< 0.001, Supplementary Figure S1D). Although miR‐195 did not correlate with VCAM‐1 (*r *= −0.118, *p* = 0.192, Supplementary Figure S1E), it was inversely associated with ICAM‐1 (*r *= −0.174, *p* = 0.043, Supplementary Figure S1F).

### 3.8 Factors relating to CHD risk

Multivariate logistic regression analyses displayed that higher miR‐26 (*p *< 0.001) and higher miR‐195 (*p *= 0.030) independently correlated with reduced CHD risk, while higher CRP (*p *= 0.009) independently correlated with increased CHD risk (Supplementary Table S1).

## 4 DISCUSSION

Lnc‐UCA1 has been recently identified as an atherosclerosis‐associated circulating lncRNA, indicating its involvement in cerebro‐cardiovascular diseases.^14^ For instance, lncRNA UCA1 increases cell proliferation and migration in VSMCs by inhibiting miR‐26a‐mediated phosphatase and tensin homolog (PTEN) expression.^15^ In addition, lnc‐UCA1 promotes cell viability, migration, and tube formation of human microvascular endothelial cell by regulating miR‐195‐mediated mitogen‐activated protein kinase kinase (MEK)/extracellular signal‐regulated kinase (ERK) and mammalian target of rapamycin (mTOR) signaling pathways.^16^ In the clinical aspect, lnc‐UCA1 is increased in chronic heart failure patients and acute ischemic stroke patients compared to that of controls,^29^, ^30^ while no published study has focused on the relation of lnc‐UCA1 with miR‐26a and miR‐195 in CHD patients, along with their correlation with disease risk, stenosis degree, and inflammation of CHD. Therefore, this study was conducted, and we discovered that lnc‐UCA1 expression tend to be increased, while miR‐26a and miR‐195 expressions were reduced in patients with CHD compared to that of controls. Also, lnc‐UCA1, miR‐26a, and miR‐195 could differentiate CHD patients from controls. There are several possible reasons which explain these findings: (a) lnc‐UCA1 promoted VSMC proliferation, which further led to increased plaque formation, thereby eventually resulted in atherosclerosis and elevated CHD risk.^15^, ^29^ (b) miR‐26a inhibited the apoptosis of endothelial cells through regulating janus kinase (JAK)/signal transducers and activators of transcription (STAT) and mitogen‐activated protein kinase (MAPK)/vascular endothelial growth factor (VEGF) pathways, which further prevented endothelium from being damage, thus led to reduced risk of CHD.^18^, ^19^ (c) miR‐195 suppressed the autophagy of endothelial progenitor cells, which further led to reduced risk of endothelium damage and less CHD risk.^21^ Furthermore, we also found that a negative correlation of lnc‐UCA1 with miR‐26a and miR‐195 in CHD patients which might be explained as that lncRNAs was able to serve as competitive endogenous RNAs by inhibiting target microRNA expression, also miR‐26a and miR‐195 might be the target of lnc‐UCA1 from previous studies; therefore, lnc‐UCA1 was negatively correlated with miR‐26a and miR‐195 in CHD patients.^30^

Several studies also investigate the correlation of lnc‐UCA1 with disease severity in cerebro‐cardiovascular disease patients, which may be utilized to monitor disease progression.^22^, ^23^ For instance, elevated lnc‐UCA1 is related to lower left ventricle ejection fraction and higher national institute of health stroke scale score in chronic heart failure patients and acute ischemic stroke patients, respectively.^22^, ^23^ While no relevant study reports the correlation of miR‐26a or miR‐195 with stenosis degree in cerebro‐cardiovascular disease patients including CHD patients. In the present study, we discovered that lnc‐UCA1 was positively correlated with Gensini score, TC, LDL‐C, and CRP in CHD patients, while miR‐26a and miR‐195 were negatively correlated with disease stenosis degree and some biochemical indexes in CHD patients, which could be explained as that (i) lnc‐UCA1 promoted oxidative stress and cell apoptosis in macrophage, which further accelerated the occurrence of atherosclerosis, therefore led to increased hyperlipidemia and advanced stenosis degree in CHD patients.^17^, ^31^ (ii) miR‐26a and miR‐195 might prevent endothelial cell apoptosis and damage as mentioned earlier, which causes reducing risk of initiating atherosclerotic events and less stenosis degree in CHD patients.^19^, ^21^

Inflammatory cytokine also plays a critical in role CHD since atherosclerosis is an inflammation‐mediated pathological event.^31^ Therefore, measuring inflammatory cytokines and exploring the correlation of lnc‐UCA1, miR‐26a, and miR‐195 with inflammatory cytokines may certainly reflect disease progression in CHD patients. From the accumulating evidence, lnc‐UCA1 promotes inflammation in the animal model of Parkinson's disease and polycystic ovary syndrome through the regulation of phosphatidylinositol 3 kinase (PI3K)/protein kinase B (AKT) pathway.^32^, ^33^ Moreover, miR‐26a reduces inflammatory cytokine production through the regulation of connective tissue growth factor in the acute lung injury.^34^ Furthermore, miR‐195 suppresses inflammatory cytokine production and oxidative stress by regulation of vascular endothelial growth factor A in acute kidney injury.^35^ In the clinical field, lnc‐UCA1 was positively correlated with T helper 17 cell (Th17 cell) ratio, IL‐6, IL‐17, and ICAM‐1 in acute ischemic stroke patients,^23^ while no relevant study reports the correlation of miR‐26a or miR‐195 in CHD patients. In the present study, we discovered that lnc‐UCA1 was positively correlated, while miR‐26a and miR‐195 were negatively correlated with inflammatory cytokines as well as cell adhesion molecules in CHD patients. These findings may possibly be due to the following reasons: (i) lnc‐UCA1 might regulate Th cell activation in endothelial cells, which resulted in an impaired immune response and thus led to increased inflammation in CHD patients^32^ (ii) miR‐26a might mediate regulatory T‐cell function and further to suppress immune response under atherosclerotic condition, thus led to reduced inflammatory cytokines in CHD patients.^36^

The measurement of lncRNA UCA1, miR‐26a, and miR‐195 might provide additional assistance for CHD diagnosis and management, while further study with a larger sample to validate our findings in CHD patients was needed. Moreover, the interaction of lncRNA UCA1 with miR‐26a and miR‐195 might be involved in CHD development, which sheds the light on future study of CHD pathogenesis and treatment. Furthermore, the detection of various polymorphisms of these noncoding RNAs might contribute to an individualized CHD management in the future. However, there were some limitations in the current study. For instance, our study did not detect the atherosclerotic plaque occurrence in CHD patients by imaging techniques, where further study could explore the correlation of lnc‐UCA1, miR‐26a, and miR‐195 with plaque occurrence in CHD patients. Moreover, the current study did not explore the correlation of these biomarkers with prognosis in CHD patients (i.e., restenosis risk, major adverse cardiac events), which could be investigated in the further study.

In conclusion, lnc‐UCA1, miR‐26a, and miR‐195 correlate with CHD risk, also they are intercorrelated and they associate with stenosis degree, cholesterol levels, inflammatory cytokines, and adhesion molecules in CHD patients.

## CONFLICT OF INTEREST

The authors declare that they have no conflicts of interest.

## Supporting information

Fig S1

Table S1

## Data Availability

Data sharing is not applicable to this article as no datasets were generated or analyzed during the current study.
