# Supplementary figures and images for: LncRNA UCA1, miR‐26a, and miR‐195 in coronary heart disease patients: Correlation with stenosis degree, cholesterol levels, inflammatory cytokines, and cell adhesion molecules
Source: J Clin Lab Anal. 2021 Dec 1;36(1):e24070. doi: 10.1002/jcla.24070 (PMC8761467; doi:10.1002/jcla.24070)

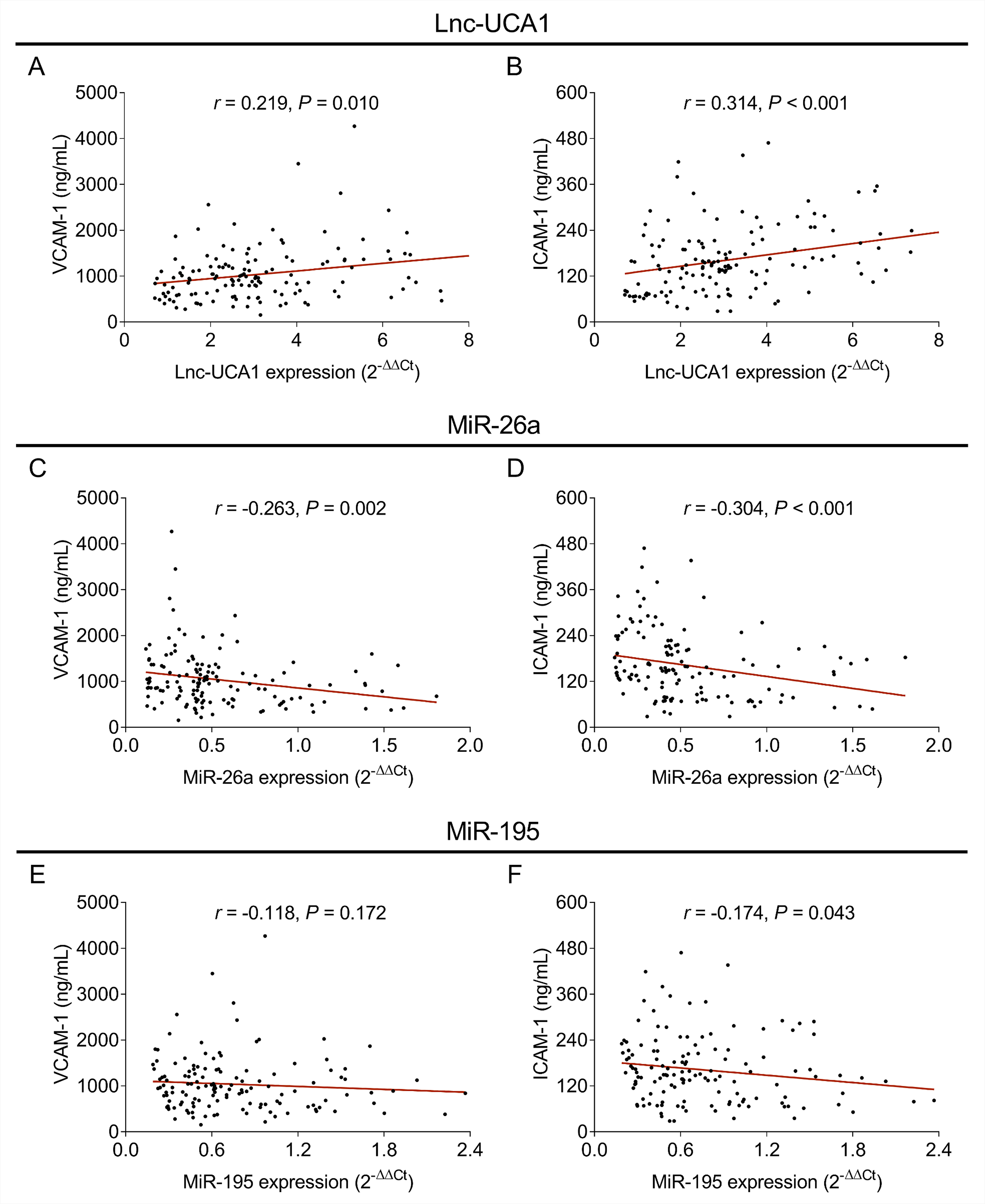

Supplement: Supplementary file 1 — Fig S1 [file JCLA-36-e24070-s002.tif]
