# Supplementary material for: LncRNA UCA1, miR‐26a, and miR‐195 in coronary heart disease patients: Correlation with stenosis degree, cholesterol levels, inflammatory cytokines, and cell adhesion molecules
Source: J Clin Lab Anal. 2021 Dec 1;36(1):e24070. doi: 10.1002/jcla.24070 (PMC8761467; doi:10.1002/jcla.24070)
Supplement: Supplementary file 2 — Table S1 [file JCLA-36-e24070-s001.docx]

**Supplementary table 1.** Factors relating diagnosis of CHD by logistic regression model analysis

| Items | *P* value | OR | 95%CI | |
| --- | --- | --- | --- | --- |
|  |  |  | Lower | Upper |
| **Multivariate logistic regression** |  |  |  |  |
| Higher miR-26a | **<0.001** | 0.077 | 0.031 | 0.189 |
| Higher miR-195 | **0.030** | 0.422 | 0.194 | 0.919 |
| DM (Yes vs. No) | 0.051 | 2.669 | 0.995 | 7.157 |
| Higher CRP | **0.009** | 1.121 | 1.029 | 1.222 |

CHD, coronary heart disease; OR, odds ratio; CI, confidence interval; miR-26a, microRNA-26a; miR-195, microRNA-195; DM, diabetes mellitus; CRP, C-reactive protein.
